# Association between Dietary Patterns and Physical Fitness among Chinese Children and Adolescents in Shaanxi Province

**DOI:** 10.3390/nu14183677

**Published:** 2022-09-06

**Authors:** Xingyue Zhang, Chuangui Mao, Yuanyuan Tan, Zijun Lu, Zheng’ao Li, Ling Zhang, Yuliang Sun, Wenfei Zhu

**Affiliations:** School of Physical Education, Shaanxi Normal University, Xi’an 710119, China

**Keywords:** dietary pattern, breakfast frequency, egg intake, dairy intake, sugared beverage intake, physical fitness, children and adolescents

## Abstract

Background: This study aims to investigate the associations between dietary patterns (breakfast, egg, dairy products, and sugared beverage intake frequencies) and physical fitness among Chinese children and adolescents in Shaanxi Province. METHODS: Data were extracted from the Chinese National Survey on Students’ Constitution and Health (CNSSCH). The study ultimately included 7305 participants (48.4% male, 51.6% female) aged 6–22 in Shaanxi Province, China. Multiple linear regression was used to examine the association of the frequency of breakfast, egg, dairy product, and sugared beverage intakes with physical fitness. RESULTS: The frequency of breakfast, egg, and dairy product intakes were all independently and positively associated with the level of physical fitness. The frequency of sugared beverage intake was negatively associated with the level of physical fitness. CONCLUSION: Healthier dietary patterns (i.e., higher breakfast, egg, and dairy product intakes and lower sugared beverage intake) were associated with greater physical fitness. Specifically, maintaining a healthy dietary pattern of breakfast, egg, and dairy product intakes can positively affect the strength and endurance performance of children and adolescents. Increased dairy product intake plays a crucial part in boosting the physical fitness total scores of children and adolescents.

## 1. Introduction

Dietary patterns are important to the nutritional statuses of diverse populations [1,2]. By following a healthy diet, one may ensure that their food consumption is balanced in terms of the kinds of foods, the quantity of the foods, and the frequency at which they eat, which not only meets their physical demands but also helps to preserve their fitness [3]. Therefore, it is crucial to cultivate healthy eating and hygiene practices throughout life. It should be noted that childhood and adolescence are significant periods during which the dietary habits that are formed will continue throughout one’s lifespan [4]. The quality of an individual’s dietary patterns, including the types and amounts of foods, nutrients, and energy that they consume, was recognized as a critical component for maximizing the potential benefits of nutrient intake [5]. Based on the Chinese Dietary Guidelines (TCDG), a regular breakfast, adequate protein intake, and a modest intake of energy-dense sugared beverages are crucial for the growth of children and adolescents [6].

A regular breakfast, as a part of a healthy diet, is associated with adequate nutritional intake, good diet quality, and physical fitness in children and adolescents [7,8]. The traditional Chinese breakfast is often rich in carbohydrates, which play an important role in meeting the needs of daily activities and can even enhance physical performance [9,10]. Beyond this, adequate protein supplements can further increase the aerobic capacity of children and adolescents [11]. Specifically, eggs and dairy products are key protein sources in the daily diet [12]. The human body needs phospholipids, cholesterol, and vitamins A and D; all of which are abundant in eggs. One study’s findings revealed that eating eggs raised the consumption of certain nutrients by 10% [13]. These nutrients include lecithin, triglycerides, and cholesterol, all of which are good for the neurological system and physical fitness and are found in egg yolks. Furthermore, research shows that supplementing with 500 mg of calcium per day can greatly improve bone density and encourage the growth of the spine and hip bones, improving physical fitness in children and adolescents [14].

Conversely, sugared beverages, such as carbonated soft drinks and fruit-flavored drinks, contain significant quantities of sugar, which raises the body’s glycemic load and exacerbates insulin sensitivity. The regular consumption of sugared beverages greatly raises a child’s or adolescent’s chance of being overweight or obese [15,16]. Several studies have proved that children and adolescents with healthier diets have better physical fitness, including muscle strength, endurance, flexibility, etc. [17,18,19,20]. Additionally, a positive association has been found between a healthy diet and cardiorespiratory fitness, speed, and agility in boys [21,22,23]. The results of these prospective studies suggest that cardiorespiratory fitness during adolescence seems to confer better development in adulthood. However, a significant proportion of children and adolescents in China continue to engage in unhealthy dietary habits, such as skipping breakfast or failing to eat a nutritious one, avoiding eggs and milk, and frequently consuming sugared beverages [24].

To our knowledge, most previous studies have been devoted to studying the connection between a balanced diet and specific types of physical fitness, such as cardiovascular and metabolic diseases [17,19,21,22]. Few studies have comprehensively analyzed the association between dietary patterns and physical fitness indicators [20,25,26]. Therefore, this study aims to investigate the associations of the frequency of breakfast, egg, dairy products, and sugared beverage intakes with physical fitness among Chinese children and adolescents in Shaanxi Province. We selected four dietary pattern variables and nine indicators of physical fitness, based on Chinese dietary guidelines. We hypothesized that (1) there were positive associations of breakfast, eggs, and dairy product intake with physical fitness in Chinese children and adolescents and (2) there was a negative association between sugared beverage intake and physical fitness in children and adolescents. The results of this study can provide directions for future intervention studies for promoting physical fitness (as measured by forced vital capacity (FVC), sit-and-reach, 1 min pull-ups/sit-ups, standing long jump, 50 m dash, 1000 m/800 m/50 m × 8 shuttle run, and 1 min rope skipping) in children and adolescents.

## 2. Materials and Methods

### 2.1. Participants

Data were extracted from the Chinese National Survey on Students’ Constitution and Health (CNSSCH). The study is the largest nationally representative survey of the health status of Chinese children and adolescents aged 6–22 years [27]. A total of 241,536 participants were randomly recruited nationwide in 2019. This project was completed during the fall semester (September–February). We collected the dietary patterns, physical activities, and physical fitness data of 11,572 school children and adolescents aged from 6 to 22 years in Shaanxi Province. All of the participants were asked to complete questionnaires and physical fitness tests. After excluding the invalid questionnaires and those with missing test items, a total of 7305 participants were included. For further analysis, the participants were divided into three academic stages, including university, middle school, and primary school. The local ethics committee approved the study at Shaanxi Normal University (202016001 2020-09).

### 2.2. Questionnaires

The participants’ dietary patterns were investigated using a survey from the report on the 2014 National Survey on Students’ Constitution and Health [28]. The questionnaires were distributed and collected uniformly and were completed by the participants independently. For the primary school students (aged from 6–12 years), the questionnaires were completed under the guidance of their teachers who had been trained in advance. This part of the questionnaire of the survey included four questions about dietary patterns:

1. In the past 7 days, how many days did you eat breakfast?
2. In the past 7 days, how many days did you eat at least 1 egg?
3. In the past 7 days, how many days did you drink at least 1 glass of milk/yogurt or soy milk?
4. In the past 30 days, how many times per day did you usually drink sugared beverages, such as cola, tea drinks, drinks with fruit juice, etc.?

For questions 1, 2, and 3, the participants were asked to select the answer that matched their diet, with eight options for the answer (options 1–8 correspond to frequencies 0–7 days, respectively). Question 4 had seven options, which corresponded to ‘none’, ‘less than 1 time per day’, ‘1 time per day’, ‘2 times per day’, ‘3 times per day’, ‘4 times per day’, and ‘5 times per day or more’ [28].

The physical activity (PA) status was obtained via the application of the same questionnaire that was used in our previous study [29]. Moderate-to-vigorous physical activity (MVPA) was calculated based on the participants’ completed PA questionnaires. This part of the questionnaire of the survey, about PA, is as below:

In the last seven days, how many of these three PA activities (light, moderate, or vigorous) did you do? What is the average number of minutes per day for each?

### 2.3. Physical Fitness Test

Following the *2019 National Student Physical Fitness and Health Research* handbook [30], we tested the participants in different grades on the corresponding variables (Table 1). The measuring personnel were strictly trained. After the tests, we calculated sex-specific and age-specific standardized values for each indicator based on the National Student Physical Fitness Standards (revised in 2014). Then, we calculated the total physical fitness scores of the participants based on the weights of each indicator in order to explain the variability between the boys and the girls and between the academic stages. The total scores consisted of standard scores and additional scores. The standard score indicators included BMI, FVC, sit-and-reach, 1 min pull-ups/sit-ups, standing long jump, 50 m dash, 1000 m/800 m/50 m × 8 shuttle run, and 1 min rope skipping. Additional scores were awarded for variables over 100 (1 min rope skipping, 1 min pull-ups/sit-ups, 50 m dash, and 1000 m/800 m/50 m × 8 shuttle run). All of the test items were finished before noon.

The participants’ flexibility was assessed by sit-and-reach. According to the changing physical capabilities of the participants’ academic stage and sex, body muscle strength was assessed by 1 min pull-ups and sit-ups. The lower body explosive strength was assessed by standing long jump. Speed and endurance were assessed by 50 m dash and time for endurance running (1000 m/800 m/50 m × 8 shuttle run), respectively. The 1 min rope skipping was used to assess coordination and agility.

### 2.4. Specifications of Physical Fitness Tests

Each participant’s BMI (kg/m^2^) was calculated as their body weight (kg) divided by their height (m) squared. The height (cm) was measured to the nearest 0.1 cm with stadiometers and the weight (kg) was measured to the nearest 0.1 kg with an electronic scale or lever scale. The participants were required to wear only light clothing and stand straight, barefoot, and at ease while being measured.

In the FVC test, the participants were asked to stand naturally, hold the tubular handle, breathe in deeply, and breathe out slowly with maximum force until they could not exhale any further. The test was performed twice with an interval of no more than 15 s between the two tests and the best result was recorded.

The 1 min pull-ups test used a high bar, which was as thick as the student’s hand could hold. The participants were asked to stand and hold the bar with their hands shoulder-width apart and then pull up. No other movements of the body were allowed. When their jaws exceeded the upper edge of the bar and they then returned to the straight arm suspension position, one completion was recorded. The participants were asked to complete as many pull-ups as possible within 1 min and the final number of completions was recorded.

The 1 min sit-ups used soft cushions and stopwatches. The participants were required to lie on their backs on the cushion with their heads in their hands and knees bent at 90°. Their partners pressed down on their ankle joints in order to immobilize the participants’ lower extremities. When a participant’s elbows touched or exceeded both knees, this was considered to be one completion. The participants were asked to complete as many sit-ups as possible within 1 min and the final number of completions was recorded.

The standing long jump was tested on flat ground. The participants were asked to stand behind the jump line with their feet apart and to push both of their feet off of the ground at the same time. We measured the vertical distance between the trailing edge of the jump line and the trailing edge of the nearest landing spot. The test was conducted three times and the best result was recorded.

In the 50 m dash and endurance running (1000 m/800 m/50 m × 8 shuttle run) tests, the participants were asked to use a standing start. For the 50 m × 8 shuttle run tests, the participants were asked to run in a counter-clockwise direction around a signpost after reaching the signpost’s placement.

The sit-and-reach tests used seated-forward bends. The participants were instructed to bend their bodies forward with their knees straight and to push the cursor forward at an even speed with the middle fingertips of both hands until they could not push any further. The test was performed twice and the examiner recorded the maximum value.

In the 1 min rope skipping test, the participants were required to adjust the length of the rope, take off on the balls of their feet, and complete the circular jumping movement in the way of “swinging on both feet”. They were asked to complete as many skips as possible within 1 min and the final number of completions was recorded.

### 2.5. Statistical Analysis

The mean (M) and standard deviation (SD) are reported for all of the quantitative variables. The data analysis was performed using SPSS 25.0 software (IBM, Chicago, IL, USA). The data of the CNSPFS were checked for Gaussian distributions using k-density plots and the extreme outliers were removed using a z-score cut-point of ±3.0. A one-way ANOVA (post hoc test: Tukey) was used to analyze the differences between the sex/academic stage groups. Multiple linear regression was used to examine the association between the breakfast, eggs, dairy products and sugary drink intakes and the students’ physical fitness. The participants’ sex, academic stage, geographic region (urban/rural) and MVPA were included as control variables. A *p*-value < 0.05 denoted statistical significance.

## 3. Results

### 3.1. The Differences in the Dietary Patterns and Physical Fitness across Different Sex/Academic Stage Groups

The study selected 7305 participants, 3534 (48.4%) of whom were boys and 3771 (51.6%) of whom were girls. There were 967 (13.2%) primary school boys, 1845 (25.3%) middle school boys, 722 (9.9%) college boys, 965 (13.2%) primary school girls, 2045 (28%) middle school girls, and 761 (10.4%) college girls (Table 2).

There were significant differences between the boys and girls in their dietary patterns, MVPA, and physical fitness (*p* < 0.05). In each sex group, a significant difference could also be found across academic stages (*p* < 0.01). As the academic stage progresses, the intake frequency of breakfast and dairy products decreased. In particular, girls had higher consumption of breakfast and dairy intake frequency. In addition, the total physical fitness scores of the girls were higher than those of the boys, which was contrary to the levels of the participants’ MVPA. At the primary school level, there were no differences between the boys and girls in the breakfast, eggs, dairy products, or sugared beverage intake frequencies, nor their BMIs. In this stage, the girls performed better than the boys in the sit-and-reach, 1 min rope skipping, and total physical fitness scores. At the middle school level, the breakfast frequency of the girls was significantly higher than that of the boys (*p* < 0.05). The egg, dairy products, and sugared beverage intake frequencies were significantly higher in the boys than in the girls (*p* < 0.01). The differences in the other physical fitness indicators between the sex groups were significant (*p* < 0.01, except for those of the 50 m × 8 shuttle run and total scores). It is worth noting that the difference in physical fitness has been increasing from middle school to college. At the college stage, there was a significant difference in the dairy intake frequency (*p* < 0.05) and highly significant differences in the BMI, FVC, sit-and-reach, standing long jump, 50 m, and total scores (*p* < 0.01).

Table 3, Table 4, Table 5 and Table 6 show the association of the frequency of eating breakfast, eggs, and dairy products in the last seven days and the number of sugared beverages ingested per day in the previous 30 days for all of the participants with their physical fitness performance.

### 3.2. The Association between Breakfast Consumption Frequency and Physical Fitness

The association between the frequency of breakfast consumption and physical fitness in children and adolescents is presented in Table 3. There was a significant positive association of the frequency of breakfast consumption with FVC and strength (as measured by 1 min pull-ups) in boys. There was also a significant positive association between the frequency of breakfast consumption and explosive strength (standing long jump) for both boys and girls. Simultaneously, the frequency of breakfast consumption had a significantly negative association with the endurance scores of boys and girls. The scores that were collected for the 50 m dash and endurance run (1000 m/800 m/50 m × 8 shuttle run) were the time (s) of running and less time indicated better performance. That is, a higher frequency of breakfast consumption was associated with a higher endurance performance for children and adolescents.

### 3.3. The Association between Egg Intake Frequency and Physical Fitness

The association between the frequency of egg intake and physical fitness in children and adolescents is presented in Table 4. The frequency of the participants’ egg intake was significantly negatively associated with the endurance running scores (1000 m/800 m/50 m × 8 shuttle run) of the children and adolescents. The frequency of the egg intake was positively associated with the scores of the 50 m dash and negatively associated with the FVC in boys. The frequency of egg intake was positively associated with flexibility (sit-and-reach) and coordination and agility (1 min rope skipping) in girls.

### 3.4. The Association between Dairy Product Intake Frequency and Physical Fitness Test

The association between the frequency of dairy product intake and physical fitness is presented in Table 5. The frequency of dairy product intake showed a significant negative association with BMI and endurance running scores (1000 m/800 m/50 m × 8 shuttle run). Also, it was positively associated with strength (1-min pull-ups, sit-ups) in girls and boys and explosive strength (standing long jump) in boys. Furthermore, more frequent dairy product intake was found to correlate with higher total scores on the physical fitness test in children and adolescents.

### 3.5. The Association between Sugared Beverage Intake Frequency and Physical Fitness Test

The association between the frequency of sugared beverage intake and physical fitness is presented in Table 6. The frequency of sugared beverage intake was negatively associated with the flexibility (sit-and-reach), endurance (1000 m/800 m/50 m × 8 shuttle run), and coordination and agility (1-min rope skipping) of boys and girls. The frequency of sugared beverage intake was negatively associated with strength (1 min pull-ups), explosive strength (standing long jump), and total scores in boys. The frequency of sugared beverage intake was also negatively associated with scores of the 50 m dash in boys, indicating that it positively affected boys’ speed performance. There was a positive association of sugared beverage intake with FVC in girls.

## 4. Discussion

Dietary patterns play a crucial role in the physical fitness of children and adolescents [4]. Our results show evidence that the frequency of breakfast, egg, and dairy product intakes were positively associated with physical fitness. In contrast, the frequency of sugared beverage intake was negatively associated with the level of physical fitness. These are findings which support our hypotheses. Specifically, maintaining higher frequencies of breakfast, egg, and dairy product intake was positively associated with strength and endurance performance in children and adolescents. Thus, this study provides guidance for children and adolescents to cultivate a healthy diet and promote their physical fitness development.

Breakfast was suggested to be the most important source of energy and nutrition throughout the day [5]. Our study found that a higher breakfast intake frequency was associated with higher endurance performance in children and adolescents. It was consistent with findings observed in the HELENA (Healthy Lifestyle in Europe by Nutrition in Adolescence) project, which showed that adolescents who consumed breakfast regularly had a healthier cardiovascular profile [31]. In this study, we also found a positive association between breakfast consumption frequency and explosive strength in children and adolescents and FVC and strength in boys. This may be related to the special dietary structure of Shaanxi, China, wherein children and adolescents consume more starches at breakfast. This dietary habit of breakfast plays a crucial role in meeting the body’s daily carbohydrate requirements [10,32]. Additionally, the tests were usually performed in the morning and the children and adolescents who consumed breakfast had more energy (sugar) to finish all of the measurements.

In our study, a higher frequency of egg intake was associated with better endurance performance in children and adolescents. A higher frequency of egg intake in boys was associated with better strength performance (1 min pull-ups). This result was consistent with that of a previous study that demonstrated that adequate protein intake is essential for improving FVC and maintaining muscle mass and strength [33]. This effect is probably due to the fact that egg and milk consumption can increase vitamin D and protein supplementation, enhancing mid-upper arm circumference for children and adolescents [34,35], which could improve their performance of the pull-ups. Our results showed that the frequency of egg intake was positively associated with speed performance (50 m dash) in children and adolescents. However, we have not found relevant evidence to explain this association. Further studies are needed to reveal this correlation.

A positive association was found between the frequency of dairy product intake and the total physical fitness scores. Dairy products provide several nutrients that are essential for physical fitness and development, such as calcium, vitamin D, and vitamin A [36]. This study showed that a higher frequency of dairy product intake was associated with better strength (1 min pull-ups/sit-ups) and endurance (1000 m/800 m/50 m × 8 shuttle run) and was positively related to explosive strength (standing long jump) for boys. Dairy products are one of the foods that are rich in vitamin D and calcium. There are several studies that have proved that vitamin D promotes calcium absorption in the body, which could promote bone growth and the maintenance of bone density and muscle strength [37,38]. As protein intake increases, amino acid availability increases and total protein oxidation increases; these are processes which may be related to better endurance performance [39].

In our findings, the frequency of the intake of sugared beverages was negatively associated with most of the measured physical fitness indicators, such as the total physical fitness scores, explosive strength (standing long jump), endurance (1000 m/800 m/50 m × 8 shuttle run), strength (1 min pull-ups/sit-ups), and coordination and agility (1 min rope skipping). For children and adolescents, excessive intake of carbohydrate-containing sugared beverages increases their caloric intake without significant additional nutritional value. In addition, high intake levels interfere with the proper balance of the body’s composition and health requirements [16]. Sugared beverages were also the primary dietary source of caffeine intake in children and adolescents [40]. The potential side effects of caffeine may pose a risk to the development of the neurological and cardiovascular systems and the body’s dependence on and addiction to it [41]. Several previous studies have reported that, in addition to energy intake, the high amount of added sugar in sugared beverages may lead to a high glycemic load and insulin response, thereby increasing the risk of obesity [42,43]. However, our results showed that sugared beverage intake was negatively associated with BMI in boys. We speculate that this may be because (1) on average, the children and adolescents did not consume many sugared beverages in the past 30 days and thus they probably had little effect on the participants’ BMIs (Mean ± SD: 1.2 ± 1.3, 1.3 ± 1.2, 1.2 ± 0.9) and (2) the types of sugared beverages and total intakes were not investigated in our questionnaires, such as energy or vitamin beverages. Further study is needed to find out the association between sugared beverages and BMI.

In our study, we found that higher academic stages in children and adolescents correlated with less frequency of breakfast and dairy product intake, total physical fitness scores, and MVPA. Previous studies have shown that breakfast quality scores decreased with progressive age in children [44]. This decrease may be influenced by a reduction in parental control in enforcing a ‘healthy’ breakfast habit. Also, it may be related to the fact that primary and middle school students lead a regular life routine, while college students become inert due to their free class time. Besides this, it has also been found that nutritional knowledge and the frequency of breakfast consumption are positively linked [45]. Therefore, schools should strengthen their regular routine and provide focused education on diet for college students in order to continuously strengthen their health awareness and thus motivate them to develop good eating habits. At the same time, universities can intervene with students’ multiple risky behaviors in order to enhance their self-control and self-management abilities so as to improve their physical fitness [45,46].

However, our study did not find an association between breakfast consumption frequency and BMI in children and adolescents. Another study showed that, in Switzerland, students who regularly ate breakfast had lower BMIs and scored better on physical fitness tests [47]. Our failure to find an association might be due to the dietary differences between China and other countries.

The advantages of this study include: (1) This was one of the first studies focusing on the association between dietary patterns and physical fitness in Chinese children and adolescents in such a large sample with diverse sex and age groups. (2) The measurements of physical fitness that are involved in this study followed the strict standards of the Chinese Dietary Guidelines (TCDG) and the Chinese National Survey on Students’ Constitution and Health (CNSSCH).

Some limitations of this study include: (1) This is a cross-sectional study which can only show the correlation between dietary patterns and physical fitness, but cannot infer a causal relationship. (2) In our study, the primary school students (aged from 6–12 years) were organized to fill in the questionnaires under the guidance of their teachers who had been trained ahead. However, the inclusion of bias was inevitable. In addition, the puberty of adolescents was not discussed in the study, which plays an important role in changes in physical performance. (3) Our study only investigated the frequency of breakfast, eggs, dairy products, and sugared beverage intakes, but not the detailed composition of the breakfast and the total energy intake. (4) Family perceptions of diet and economic status are important influences on dietary patterns. These factors could be included as control variables in future studies, which could better explain the results.

## 5. Conclusions

Dietary patterns are essential factors that are related to the physical fitness of children and adolescents. The frequency of the participants’ breakfast, egg, and dairy product intakes were positively associated with their physical fitness. The frequency of the participants’ sugared beverage intake was negatively associated with their physical fitness. Especially, increased dairy product consumption plays a crucial part in boosting the total physical fitness scores of children and adolescents. However, the breakfast and dairy products consumption frequencies and total physical fitness scores in children and adolescents decreased as their academic stage increased. Accordingly, nutrition education should be strengthened for children and adolescents and their parents. Schools should also help children and adolescents to establish healthy eating behaviors. At the same time, children and adolescents should also enhance their self-control and self-management abilities and maintain their healthy breakfasts, eggs, and dairy product intake frequencies and lower their consumption of sugared beverages. The implementation of these changes can help children and adolescents to maintain excellent physical fitness.

## Figures and Tables

**Table 1:** Variable and weight.

Tested Object	Variable	Weight (%)
Grades 1 and 2	Body mass index	15
	Force vital capacity	15
	50 m dash	20
	Sit-and-reach	30
	1 min rope skipping	20
Grades 3 and 4	Body mass index	15
	Force vital capacity	15
	50 m dash	20
	Sit-and-reach	20
	1 min rope skipping	20
	1 min sit-ups	10
Grades 5 and 6	Body mass index	15
	Force vital capacity	15
	50 m dash	20
	Sit-and-reach	10
	1 min rope skipping	10
	1 min sit-ups	20
	50 m × 8 shuttle run	10
All grades in high school and college	Body mass index	15
	Force vital capacity	15
	50 m dash	20
	Sit-and-reach	10
	Standing long jump	10
	1 min pull-ups(boys)/sit-ups(girls)	10
	1000 m(boys)/800 m(girls)	20

Body mass index (BMI) = weight(kg)/height^2^(m^2^); e.g., (Grades 1 & 2) Total scores = BMI × 0.15 + FVC × 0.15 + 50 m × 0.2 + sit and reach × 0.3 + 1 min rope skipping × 0.2+ (additional scores).

**Table 2:** Sample descriptive data according to the school period.

Variables	Boys				Girls			
	Primary School	Middle School	College		Primary School	Middle School	College	
	*n* = 967	*n* = 1845	*n* = 722	*p*	*n* = 965	*n* = 2045	*n* = 761	*p*
	13.20%	25.30%	9.90%		13.20%	28.00%	10.40%	
Number of breakfast days in the past 7 days	6.5 ± 1.3	5.6 ± 1.3	4.4 ± 2.3	<0.001	6.6 ± 1.0	5.8 ± 1.7 *	5.4 ± 1.9	<0.001
Number of days eating eggs in the past 7 days	3.8 ± 2.3	2.5 ± 2.2	2.7 ± 2.3	<0.001	3.9 ± 2.2	2.0 ± 2.0 **	2.5 ± 2.1	<0.001
Number of days milk/yogurt or soy milk was consumed in the past 7 days	4.7 ± 2.4	3.6 ± 2.4	3.1 ± 2.3	<0.001	4.8 ± 2.3	3.4 ± 2.3 **	3.4 ± 2.1 *	<0.001
Number of sugared beverages per day in the past 30 days	1.2 ± 1.3	1.3 ± 1.2	1.2 ± 0.9	0.001	1.1 ± 1.1	1.1 ± 0.9 **	1.0 ± 0.7 **	0.005
Height (cm)	141.0 ± 8.8	168.9 ± 7.2	173.3 ± 5.6	<0.001	142.3 ± 9.3 **	158.8 ± 5.5 **	160.8 ± 5.3 **	<0.001
Weight (kg)	33.0 ± 6.3	57.8 ± 10.7	66.5 ± 8.9	<0.001	33.8 ± 7.0 **	51.9 ± 7.7 **	54.4 ± 7.1 **	<0.001
BMI (kg/m^2^)	16.5 ± 1.9	20.9 ± 3.1	22.1 ± 2.8	<0.001	16.5 ± 2.0	20.6 ± 2.7 **	21.0 ± 2.5 **	<0.001
FVC (mL)	1719.3 ± 470.6	3395.0 ± 737.3	4278.8 ± 775.6	<0.001	1592.6 ± 417.6 **	2375.2 ± 459.5 **	2779.0 ± 517.8 **	<0.001
Sit-and-reach (cm)	4.5 ± 5.2	8.3 ± 6.7	12.3 ± 6.3	<0.001	8.8 ± 5.7 **	11.5 ± 6.1 **	16.0 ± 6.0 **	<0.001
1 min pull-ups(boys)/sit-ups(girls) ^a.^	29.0 ± 9.6	1.9 ± 2.6	5.1 ± 5.0	<0.001	25.6 ± 9.8 **	27.5 ± 8.6	31.4 ± 9.3	<0.001
Standing long jump (cm) ^b.^		202.4 ± 27.8	221.5 ± 21.8	<0.001		152.2 ± 20.5 **	162.3 ± 17.3 **	<0.001
50 m dash (s)	9.8 ± 1.0	7.9 ± 0.7	7.7 ± 0.6	<0.001	10.2 ± 1.0 **	9.6 ± 0.9 **	9.7 ± 0.8 **	<0.001
1000 m(boys)/800 m(girls)/50 m × 8 shuttle run test (s) ^c.^	118.4 ± 11.3	264.7 ± 29.8	259.6 ± 29.3	<0.001	124.2 ± 11.0 **	254.9 ± 28.7	259.8 ± 26.0	<0.001
1 min rope skipping ^d.^	72.9 ± 37.9				84.1 ± 32.9 **			<0.001
Total physical fitness scores	69.3 ± 7.1	67.4 ± 8.5	66.7 ± 8.4	<0.001	70.9 ± 6.8 **	69.0 ± 9.7	68.2 ± 9.5 **	<0.001
MVPA	456.2 ± 542.1	302.6 ± 284.2	223.8 ± 221.8	<0.001	401.3 ± 463.4 *	214.6 ± 183.3 **	169.0 ± 191.5 **	<0.001

Data expressed as mean ± SD; *p*: the statistical significance of the academic period; *: differences between boys and girls in the same period; * indicates *p* < 0.05, ** indicates *p* < 0.01. Primary school boys were tested with 1 min sit-ups (denoted by ^a^). All high school and college grades were tested with the standing long jump (as indicated by ^b^). Primary school students in grades 5 and 6 were tested 50 m × 8 shuttle run (as indicated by ^c^). All primary school grade students were tested with 1 min rope skipping (as indicated by ^d^). BMI: body mass; FVC: forced vital capacity; MVPA: moderate-to-vigorous physical activity.

**Table 3:** Association between frequency of breakfast consumption and physical fitness in children and adolescents.

	Boys (*n* = 3534)		*p*	Girls (*n* = 3771)		*p*
	B	SE		B	SE	
In the past 7 days, how many days did you eat breakfast?						
BMI (kg/m^2^)	0.000	0.023	0.985	−0.024	0.026	0.363
FVC (mL)	12.746	5.766	0.027	−6.475	4.634	0.162
Sit-and-reach (cm)	0.070	0.054	0.197	0.114	0.060	0.056
1 min pull-ups(boys)/sit-ups(girls) ^a.^	0.206	0.091	0.023	−0.009	0.090	0.924
Standing long jump (cm) ^b.^	1.160	0.230	<0.001	0.653	0.206	0.002
50 m dash (s)	−0.004	0.008	0.568	0.013	0.009	0.155
1000 m(boys)/800 m(girls)/50 m × 8 shuttle run test (s) ^c.^	−1.790	0.459	<0.001	−3.873	0.427	<0.001
1 min rope skipping ^d.^	0.757	0.868	0.383	0.932	0.936	0.320
Total scores	0.057	0.072	0.431	0.094	0.089	0.288

BMI: body mass; FVC: forced vital capacity. ^a^: this test (1 min sit-ups) was applied to primary school boys. ^b^: this test was applied to all high school and college grades. ^c^: this test (50 m × 8 shuttle run) was applied to primary school students in grades 5 and 6. ^d^: this test was applied to all primary school grade students.

**Table 4:** Association between frequency of egg intake and physical fitness in children and adolescents.

	Boys (*n* = 3534)		*p*	Girls (*n* = 3771)		*p*
	B	SE		B	SE	
In the past 7 days, how many days did you eat at least 1 egg?						
BMI (kg/m^2^)	−0.012	0.020	0.549	−0.117	0.020	<0.001
FVC (mL)	−14.393	4.881	0.003	−5.608	3.539	0.113
Sit-and-reach (cm)	−0.028	0.046	0.543	0.153	0.046	0.001
1 min pull-ups(boys)/sit-ups(girls) ^a.^	0.851	0.076	<0.001	0.122	0.069	0.077
Standing long jump (cm) ^b.^	0.359	0.222	0.106	−0.044	0.184	0.811
50 m dash (s)	0.018	0.006	0.006	0.026	0.007	<0.001
1000 m(boys)/800 m(girls)/50 m × 8 shuttle run test (s) ^c.^	−4.344	0.393	<0.001	−4.791	0.332	<0.001
1 min rope skipping ^d.^	0.241	0.486	0.621	1.164	0.429	0.007
Total scores	0.056	0.061	0.357	0.107	0.068	0.115

BMI: body mass; FVC: forced vital capacity. ^a^: this test (1 min sit-ups) was applied to primary school boys. ^b^: this test was applied to all high school and college grades. ^c^: this test (50 m × 8 shuttle run) was applied to primary school students in grades 5 and 6. ^d^: this test was applied to all primary school grade students.

**Table 5:** Association between frequency of dairy product intake and physical fitness in children and adolescents.

	Boys (*n* = 3534)		*p*	Girls (*n* = 3771)		*p*
	B	SE		B	SE	
In the past 7 days, how many days did you drink at least 1 glass of milk/yogurt or soy milk?						
BMI (kg/m^2^)	−0.042	0.019	0.027	−0.073	0.019	<0.001
FVC (mL)	−6.372	4.668	0.172	−3.208	3.347	0.338
Sit-and-reach (cm)	−0.054	0.044	0.223	0.023	0.043	0.587
1 min pull-ups(boys)/sit-ups(girls) ^a.^	0.513	0.073	<0.001	0.246	0.065	<0.001
Standing long jump (cm) ^b.^	0.853	0.206	<0.001	0.309	0.164	0.060
50 m dash (s)	−0.006	0.006	0.296	0.002	0.007	0.802
1000 m(boys)/800 m(girls)/50 m × 8 shuttle run test (s) ^c.^	−3.470	0.376	<0.001	−3.741	0.314	<0.001
1 min rope skipping ^d.^	0.343	0.488	0.482	0.176	0.436	0.686
Total scores	0.142	0.058	0.015	0.222	0.064	0.001

BMI: body mass; FVC: forced vital capacity. ^a^: this test (1 min sit-ups) was applied to primary school boys. ^b^: this test was applied to all high school and college grades. ^c^: this test (50 m × 8 shuttle run) was applied to primary school students in grades 5 and 6. ^d^: this test was applied to all primary school grade students.

**Table 6:** Association between frequency of sugared beverage intake and physical fitness in children and adolescents.

	Boys (*n* = 3534)		*p*	Girls (*n* = 3771)		*p*
	B	SE		B	SE	
In the past 30 days, how many times a day did you usually drink sugared beverages, such as cola, tea drinks, drinks with fruit juice, etc.?						
BMI (kg/m^2^)	−0.083	0.039	0.034	0.020	0.045	0.655
FVC (mL)	−1.703	9.593	0.859	22.071	8.036	0.006
Sit-and-reach (cm)	−0.212	0.090	0.019	−0.221	0.104	0.033
1 min pull-ups(boys)/sit-ups(girls) ^a.^	−0.895	0.152	<0.001	−0.298	0.157	0.057
Standing long jump (cm) ^b.^	−1.036	0.440	0.019	−0.467	0.424	0.271
50 m dash (s)	−0.048	0.013	<0.001	0.000	0.016	1.000
1000 m(boys)/800 m(girls)/50 m × 8 shuttle run test (s) ^c.^	5.021	0.807	<0.001	4.605	0.831	<0.001
1 min rope skipping ^d.^	−2.641	0.892	0.003	−1.845	0.847	0.030
Total scores	−0.261	0.121	0.031	−0.198	0.154	0.199

BMI: body mass; FVC: forced vital capacity. ^a^: this test (1 min sit-ups) was applied to primary school boys. ^b^: this test was applied to all high school and college grades. ^c^: this test (50 m × 8 shuttle run) was applied to primary school students in grades 5 and 6. ^d^: this test was applied to all primary school grade students.

## Data Availability

The data that are presented in this study are available on request from the corresponding author. The data are not publicly available for reasons of confidentiality.
